# Comparison of treatment outcomes between squamous cell carcinoma and adenocarcinoma of cervix after definitive radiotherapy or concurrent chemoradiotherapy

**DOI:** 10.1186/s13014-018-1197-5

**Published:** 2018-12-17

**Authors:** Ke Hu, Weiping Wang, Xiaoliang Liu, Qingyu Meng, Fuquan Zhang

**Affiliations:** Department of Radiation Oncology, Peking Union Medical College Hospital, Chinese Academy of Medical Sciences and Peking Union Medical College, No.1 Shuaifuyuan Wangfujing, Dongcheng District, Beijing, 100730 China

**Keywords:** Adenocarcinoma, Squamous cell carcinoma, Cervical cancer, Radiotherapy, Concurrent chemoradiotherapy

## Abstract

**Background:**

Concurrent chemoradiotherapy (CCRT) is effective in the treatment of locally advanced cervical squamous cell carcinoma (SCC). However, whether treatment outcomes of cervical adenocarcinoma are equivalent to SCC after CCRT has been a topic of debate.

**Methods:**

Medical records of cervical cancer patients treated with definitive radiotherapy or CCRT in our institute from January 2011 to December 2014 were reviewed. Patients were treated with intensity modulated radiation therapy combined with intracavitary brachytherapy. Weekly cisplatin was the first line regimen of concurrent chemotherapy. The treatment outcomes of patients with SCC and adenocarcinoma were compared with a multivariate Cox regression model, and log-rank method before and after propensity score matching (1:1).

**Results:**

A total of 815 patients with stage IB-IVA cervical cancer were included, with 744 patients in the SCC group and 71 patients in adenocarcinoma group. The median follow-up period was 36.2 months (range, 1.0–76.2 months). The 3-year overall survival (OS), disease-free survival (DFS), pelvic control and distant control rates of patients in the SCC group and adenocarcinoma group were 85.2 and 75.4% (*p* = 0.005), 77.5 and 57.3% (*p* < 0.001), 89.0 and 74.0% (*p* = 0.001) and 86.0 and 74.4% (*p* = 0.011), respectively. After multivariate analysis, histology was an independent factor of OS (*p* = 0.003), DFS (*p* < 0.001), pelvic control (*p* = 0.002) and distant control (*p* = 0.003). With propensity score matching, 71 pairs of patients were selected. After matching, the OS (*p* = 0.017), DFS (*p* = 0.001), pelvic control (*p* = 0.015) and distant control (*p* = 0.009) of patients with adenocarcinoma were poorer than those of patients with SCC. In subgroup analysis, patients with adenocarcinoma had significantly worse OS and DFS compared with patients with SCC, regardless of treatment with radiotherapy alone or CCRT.

**Conclusion:**

The present study demonstrated that patients with adenocarcinoma of the cervix had poorer OS and DFS than patients with SCC, regardless of treatment with radiotherapy alone or CCRT. New treatment approaches should be considered for cervical adenocarcinoma.

## Background

Cervical cancer is one of the most common gynaecological cancers in women. It was estimated that there were 13,240 new cases and 4170 deaths in United States in 2018 [1]. Squamous cell carcinoma (SCC) accounts for approximately 70% of all cervical cancer and adenocarcinoma (AC) accounts for approximately 20% [2]. With screening and human papilloma virus vaccines, the incidence of cervical cancer had significantly decreased in recent years in developed countries [2–4]. However, incidence of AC of the cervix has increased in past decades. The age-adjusted AC incidence rates increased 0.5 to 3% per annum in Europe [5]. The probable reason is that, compared with cervical SCC, cervical cytologic screening methods are less effective for cervical AC [6].

At present, there is no difference in treatments between SCC and AC of the cervix. However, clinical characteristics and prognosis of cervical AC differ from SCC. Patients with AC have been reported to be younger, more often white, more commonly diagnosed while in the early stages and more likely to have metastatic lymph nodes (MLNs) than patients with SCC [7, 8]. Whether patients with different histological subtype have different survival outcomes remains a topic of debate. In some previous studies, for early stage cervical cancer patients treated with hysterectomy and lymphadenectomy, cervical AC exhibited equivalent survival to SCC [9–11]. In some other studies, after radical hysterectomy and lymph node dissection, AC was associated with a poor survival, compared with SCC [12–14].

Currently, the standard treatment approach for locally advanced cervical cancer patients is definitive concurrent chemoradiotherapy (CCRT), regardless of the histological subtype of the disease. Studies on the prognostic significance of AC in cervical cancer patients treated with CCRT are limited, and the results are conflicting [7, 15–18]. In a study from Japan, patients with AC or adenosquamous carcinoma (ASC) of the cervix experienced worse overall survival (OS) (*p* = 0.004) and progression-free survival (PFS) (*p* = 0.002) than patients with SCC of the cervix after definitive radiotherapy. AC/ASC was also an independent prognostic factor of PFS in multivariate analysis (*p* = 0.031) [15]. A study based on the Korea National Cancer Incidence Database showed that the survival of patients with cervical AC improved after the introduction of concurrent chemotherapy. However, AC was still associated with worse OS compared with SCC for patients treated with CCRT (*p* = 0.003) [17]. Rose et al. retrospectively analysed 1671 cervical cancer patients (1489 patients with SCC and 182 patients with AC/ASC) in five Gynaecologic Oncology Group (GOG) trials, and found that when treated with radiotherapy alone, AC/ASC was associated with poor OS (*p* = 0.0449). However, when patients were treated with cisplatin-based CCRT, the OS (*p* = 0.459) and PFS (*p* = 0.315) were similar among patients with SCC and AC/ASC [7]. In the study of Katanyoo et al., 141 patients with AC were matched with 282 patients with SCC. After radiotherapy/CCRT, the complete response (CR) rates in patients with AC/ASC and SCC were 86.5 and 94.7%, respectively (*p* = 0.004). However, the 5-year OS was similar, 59.9% in patients with AC and 61.7% in patients with SCC (*p* = 0.191) [18].

In the present study, we reviewed cervical patients treated with radiotherapy or CCRT in our institute and analysed the prognostic significance of AC.

## Methods

### Patients

Medical records of cervical cancer patients treated with definitive radiotherapy or CCRT in our institute from January 2011 to December 2014 were reviewed. The inclusive criteria included the following: biopsy confirmed cervical AC or SCC; the International Federation of Gynaecology and Obstetrics (FIGO) stage IB-IVA; no evidence of distant metastases; treated with definitive radiotherapy or CCRT. Patients with all histological types except SCC and AC, including ASC, undifferentiated carcinoma, neuroendocrine carcinoma, sarcoma, lymphoma and other scarce histology, were excluded from this study.

### Treatment

Patients were treated with definitive intensity modulated radiation therapy (IMRT) and intracavitary brachytherapy (ICBT). The clinical target volume (CTV) and gross tumour volume (GTVnd) were contoured on CT simulation slices. The CTV included the cervix, uterus, parametrium, upper part of the vagina and pelvic lymph node regions. For patients with para-aortic MLNs, or with high risk of para-aortic lymph node failure, the para-aortic lymph node region was also included in the CTV [19, 20]. GTVnd covered the regional MLNs. A margin of 8 to 10 mm was added to the CTV in all directions and an additional 5 to 10 mm margin to the uterus and cervix to create the planning target volume (PTV). A 5-mm margin was given to GTVnd to generate planning gross tumour volume (PGTVnd). A dose of 50.4 Gy in 28 fractions was prescribed to the PCTV with IMRT, and the PGTVnd were simultaneously boosted to 59–61 Gy. For patients with stage IIIB disease, an additional dose of 10 Gy in 5 fractions was delivered to the parametrium in our institute. Daily megavoltage computed tomography (MVCT) or weekly cone-beam CT/CT-on-rail were used for image guidance.

ICBT was delivered with iridium-192, with 30 to 36 Gy in 5 to 7 fractions prescribed to point A. The details of radiotherapy were described previously [21, 22].

The first line regimen of concurrent chemotherapy was cisplatin 40 mg/m^2^ weekly. Patients with renal dysfunction underwent paclitaxel weekly. Part of patients with AC of the cervix received paclitaxel or paclitaxel plus cisplatin.

### Patient follow-up

Patients had a follow-up evaluation 1 month after treatment. After that, patients received follow-up examinations every 3 months in the first 2 years, every 6 months in years 3 to5 and once a year thereafter. SCC antigen, chest/abdomen CT and pelvic MRI were conducted regularly in follow-up. Positron emission tomography (PET)-CT was used for patients with suspected recurrence or metastasis.

### Statistics

The endpoints included OS, disease-free survival (DFS), pelvic control and distant control. The baseline characteristics between patients in the SCC and AC groups were compared with chi-squared (χ2) or continuity correction tests, as appropriate. Univariate analysis and multivariate analysis were performed with a Cox proportional hazard regression model. As the baseline characteristics were significantly different between two groups, propensity score matching was performed with a ratio of 1:1. The matching covariate included para-aortic MLNs, pelvic MLNs and concurrent chemotherapy. OS, DFS, pelvic control and distant control were estimated using the Kaplan-Meier method and compared between the SCC and AC groups using the log-rank method before and after matching. In subgroup analysis, the Cox regression model or log-rank methods were used to compare the survival between subgroups. Statistical analyses were performed with SPSS (version 22.0; SPSS, Inc., Chicago, IL, USA). Two-sided *p* < 0.05 was considered statistically significant.

## Results

Between January 2011 and December 2014, there were 833 patients with stage IB-IVA cervical cancer treated with definitive radiotherapy or CCRT in our institute. Nine patients with ASC, seven patients with undifferentiated carcinoma, and two patients with neuroendocrine carcinoma were excluded. Finally, 815 were patients enrolled in this study. Of them, there were 744 patients (91.3%) with SCC and 71 patients (8.7%) with AC. The detailed characteristics of patients in the SCC and AC groups are shown in Table 1. More patients in the AC group had para-aortic MLNs (5.9% in SCC group and 14.1% in AC group, *p* = 0.008), and more patients in the SCC group had pelvic MLNs (29.2% in SCC group and 19.7% in AC group, *p* = 0.091). The other variables, including age, FIGO stage, primary tumour size, common iliac MLNs, number of pelvic MLNs and CCRT were similar between two groups.

**Table 1:** Characteristics of cervical cancer patients with SCC and AC before and after matching

Characteristics	Before matching			After matching		
	SCC (*n* = 744)	AC (*n* = 71)	p	SCC (*n* = 71)	AC (*n* = 71)	p
Age (ys)						
< 65	663 (89.1%)	64 (90.1%)	0.790	66 (93.0%)	64 (90.1%)	0.546
≥ 65	81 (10.9%)	7 (9.9%)		5 (7.0%)	7 (9.9%)	
FIGO stage						
Stage I	92 (12.4%)	7 (9.9%)	0.359	3 (4.2%)	7 (9.9%)	0.403
Stage II	505 (67.9%)	54 (76.1%)		56 (78.9%)	54 (76.1%)	
Stage III-IVA	147 (19.8%)	10 (14.1%)		12 (16.9%)	10 (14.1%)	
Primary tumour size						
< 4 cm	287 (38.6%)	29 (40.8%)	0.708	28 (39.4%)	29 (40.8%)	0.864
≥ 4 cm	457 (61.4%)	42 (59.2%)		43 (60.6%)	42 (59.2%)	
Para-aortic MLNs						
Yes	44 (5.9%)	10 (14.1%)	0.008	10 (14.1%)	10 (14.1%)	1.000
No	700 (94.1%)	61 (85.9%)		61 (85.9%)	61 (85.9%)	
Pelvic MLNs						
Yes	217 (29.2%)	14 (19.7%)	0.091	14 (19.7%)	14 (19.7%)	1.000
No	527 (70.8%)	57 (80.3%)		57 (80.3%)	57 (80.3%)	
Common iliac MLNs						
Yes	56 (7.5%)	6 (8.5%)	0.779	7 (9.9%)	6 (8.5%)	0.771
No	688 (92.5%)	65 (91.5%)		64 (90.1%)	65 (91.5%)	
Number of pelvic MLNs						
0–3	705 (94.8%)	68 (95.8%)	0.711	65 (91.5%)	68 (95.8%)	0.491
≥ 4	39 (5.2%)	3 (4.2%)		6 (8.5%)	3 (4.2%)	
Concurrent chemotherapy						
Yes	613 (82.4%)	60 (84.5%)	0.654	56 (78.9%)	60 (84.5%)	0.385
No	131 (17.6%)	11 (15.5%)		15 (21.1%)	11 (15.5%)	

*AC* adenocarcinoma, *FIGO* the International Federation of Gynaecology and Obstetrics, *MLNs* metastatic lymph nodes, *SCC* squamous cell carcinoma

### Survivals of patients in SCC and AC groups

**Fig. 1:**
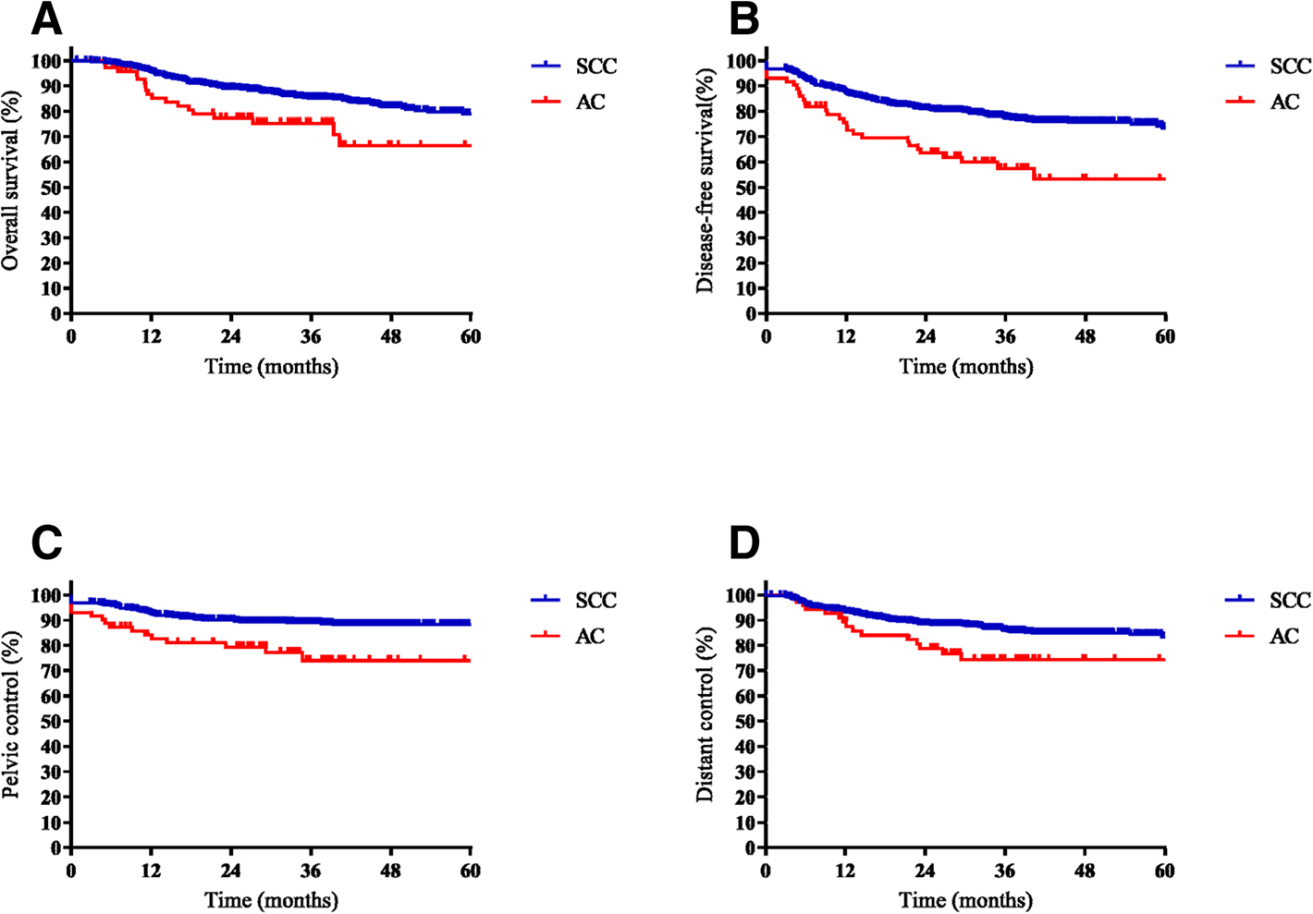
The overall survival (OS, **a**), disease-free survival (DFS, **b**), pelvic control (**c**) and distant control (**d**) of patients with squamous cell carcinoma (SCC) and adenocarcinoma (AC) of the cervix

The median follow-up period was 36.2 months (range, 1.0–76.2 months) for the whole cohort, with 37.3 months (range, 1.0–76.2 months) in the SCC group and 28.8 months (range, 5.1–68.8 months) in the AC group (*p* < 0.001). The 3-year OS, DFS, pelvic control and distant control rates in the SCC and AC groups were 85.2 and 75.4% (*p* = 0.005), 77.5 and 57.3% (*p* < 0.001), 89.0 and 74.0% (*p* = 0.001) and 86.0 and 74.4% (*p* = 0.011), respectively. The OS, DFS, pelvic control and distant control curves of patients treated with SCC and AC are shown in Fig. 1.

### Univariate and multivariate analysis

After univariate analysis (Table 2), histology was a significant factor of OS (*p* = 0.006), DFS (*p* < 0.001), pelvic control (*p* = 0.002) and distant control (*p* = 0.013). Significant factors in univariate analysis were analysed with multivariate analysis. As shown in Table 3, histology remained significant in predicting OS (hazard ratio, HR 2.21, 95% confidence interval, CI 1.31–3.74, *p* = 0.003), DFS (HR 2.37, 95% CI 1.57–3.56, *p* < 0.001), pelvic control (HR 2.40, 95% CI 1.36–4.22, *p* = 0.002) and distant control (HR 2.27, 95% CI 1.31–3.92, *p* = 0.003) after multivariate analysis.

**Table 2:** Results of univariate analysis for OS, DFS, pelvic control and distant control

Variables	OS		DFS		Pelvic control		Distant control	
	HR (95% CI)	p	HR (95% CI)	p	HR (95% CI)	p	HR (95% CI)	p
Age (< 65 vs ≥65)	2.01 (1.29–3.13)	0.002	1.38 (0.92–2.07)	0.124	0.99 (0.51–1.90)	0.968	1.20 (0.69–2.11)	0.516
Histology (SCC vs AC)	2.01 (1.22–3.31)	0.006	2.21 (1.49–3.25)	< 0.001	2.33 (1.36–3.99)	0.002	1.96 (1.15–3.32)	0.013
FIGO stage (I, II and III-IVA)	2.24 (1.65–3.06)	< 0.001	1.96 (1.52–2.52)	< 0.001	1.87 (1.31–2.69)	0.001	1.76 (1.26–2.45)	0.001
Tumour size (< 4 cm vs ≥4 cm)	2.74 (1.80–4.17)	< 0.001	2.43 (1.74–3.39)	< 0.001	2.60 (1.59–4.26)	< 0.001	2.20 (1.44–3.36)	< 0.001
Para-aortic MLNs (No vs Yes)	6.09 (4.05–9.15)	< 0.001	5.10 (3.55–7.34)	< 0.001	6.66 (4.18–10.60)	< 0.001	3.05 (1.77–5.26)	< 0.001
Pelvic MLNs (No vs Yes)	3.21 (2.28–4.50)	< 0.001	2.90 (2.20–3.84)	< 0.001	3.23 (2.16–4.82)	< 0.001	2.63 (1.82–3.79)	< 0.001
Common iliac MLNs (No vs Yes)	5.68 (3.83–8.43)	< 0.001	4.17 (2.91–5.98)	< 0.001	3.55 (2.12–5.93)	< 0.001	4.20 (2.64–6.70)	< 0.001
Number of pelvic MLNs (continuous)	1.29 (1.24–1.35)	< 0.001	1.27 (1.22–1.32)	< 0.001	1.25 (1.19–1.32)	< 0.001	1.22 (1.15–1.28)	< 0.001
Concurrent chemotherapy (No vs Yes)	0.67 (0.45–0.99)	0.046	0.71 (0.51–0.99)	0.044	0.72 (0.44–1.16)	0.178	0.83 (0.53–1.32)	0.433

*AC* adenocarcinoma, *CI* confidence interval, *FIGO* the International Federation of Gynaecology and Obstetrics, *HR* hazard ratio, *MLNs* metastatic lymph nodes, *SCC* squamous cell carcinoma

**Table 3:** Results of multivariate analysis for OS, DFS, pelvic control and distant control

Variables	OS		DFS		Pelvic control		Distant control	
	HR	p	HR	P	HR	P	HR	P
Age (< 65 vs ≥65)	2.07 (1.27–3.36)	0.004						
Histology (SCC vs AC)	2.21 (1.31–3.74)	0.003	2.37 (1.57–3.56)	< 0.001	2.40 (1.36–4.22)	0.002	2.27 (1.31–3.92)	0.003
FIGO stage (I, II and III-IVA)	1.84 (1.33–2.56)	< 0.001	1.56 (1.20–2.04)	0.001	1.41 (0.96–2.06)	0.079	1.46 (1.03–2.07)	0.036
Tumour size (< 4 cm vs ≥4 cm)	2.00 (1.28–3.14)	0.002	1.80 (1.27–2.54)	0.001	1.81 (1.08–3.03)	0.024	1.70 (1.10–2.65)	0.018
Para-aortic MLNs (No vs Yes)	1.28 (0.69–2.40)	0.434	1.40 (0.85–2.33)	0.188	2.42 (1.25–4.68)	0.008	0.73 (0.35–1.56)	0.420
Pelvic MLNs (No vs Yes)	1.43 (0.91–2.24)	0.125	1.54 (1.08–2.22)	0.018	1.62 (0.97–2.71)	0.065	1.49 (0.93–2.38)	0.097
Common iliac MLNs (No vs Yes)	1.30 (0.72–2.35)	0.392	1.08 (0.65–1.78)	0.773	0.69 (0.34–1.40)	0.304	1.67 (0.88–3.17)	0.119
Number of pelvic MLNs (continuous)	1.16 (1.07–1.26)	0.001	1.14 (1.06–1.23)	0.001	1.13 (1.03–1.25)	0.009	1.13 (1.02–1.25)	0.018
Concurrent chemotherapy (No vs Yes)	0.85 (0.55–1.29)	0.440	0.72 (0.51–1.01)	0.059				

*AC* adenocarcinoma, *CI* confidence interval, *FIGO* the International Federation of Gynaecology and Obstetrics, *HR* hazard ratio, *MLNs* metastatic lymph nodes, *SCC* squamous cell carcinoma

### Propensity score matching

To balance the basic characteristics, 71 cervical patients with AC were matched with 71 patients with SCC. The basic characteristics were similar between two groups (Table 1).

After matching, the 3-year OS, DFS, pelvic control and distant control rates of patients in the SCC and AC groups were 86.4 and 75.4% (*p* = 0.017), 82.5 and 57.3% (*p* = 0.001), 91.2 and 74.0% (*p* = 0.015) and 91.1 and 74.4% (*p* = 0.009), respectively.

### Subgroup analysis

In 142 patients treated with definitive radiotherapy alone, 11 patients had AC and 131 patients had SCC. The OS (HR 2.64, 95% CI 1.01–6.87, *p* = 0.047), DFS (HR 3.46, 95% CI 1.60–7.46, *p* = 0.002) and distant control (HR 3.22, 95% CI 1.09–9.51, *p* = 0.035) of patients with AC were worse than patients with SCC. The pelvic control (HR 2.12, 95% CI 0.62–7.20, *p* = 0.229) was not significantly different. As for 673 patients treated with CCRT (60 patients with AC and 613 patients with SCC), AC was associated with worse OS (HR 1.85, 95% CI 1.03–3.31, *p* = 0.039), DFS (HR 1.97, 95% CI 1.25–3.09, *p* = 0.003) and pelvic control (HR 2.42, 95% CI 1.33–4.41, *p* = 0.004). The distant control was not significantly different between the two groups (HR 1.73, 95% CI 0.95–3.18, *p* = 0.076).

Of the 815 patients, 96 patients experienced pelvic failure. In the other 719 patients (55 patients with AC and 664 patients with SCC) without pelvic recurrence, patients with AC also had poorer DFS (HR 2.13, 95% CI 1.21–3.74, *p* = 0.009) and distant control (HR 1.98, 95% CI 1.08–3.64, *p* = 0.027) than those with SCC. The OS (HR 1.52, 95% CI 0.66–3.54, *p* = 0.329) was similar in these two groups.

In 71 patients with AC of the cervix, 60 patients were treated with CCRT, with 46 patients receiving cisplatin, 6 patients receiving paclitaxel and 8 patients receiving paclitaxel plus cisplatin. The 3-year OS, DFS, pelvic control and distant control rates of AC patients treated cisplatin and paclitaxel/paclitaxel plus cisplatin were 78.5 and 84.6% (*p* = 0.819), 54.0 and 85.1% (*p* = 0.163), 72.1 and 85.1% (*p* = 0.149) and 71.8 and 85.1% (*p* = 0.425).

## Discussion

In the present study, we found that patients with AC had worse survival, compared with patients with SCC. In the SCC and AC groups, the 3-year OS rates were 85.4 and 75.4%, respectively (*p* = 0.005), and the 3-year DFS was 77.5 and 57.3%, respectively (*p* < 0.001). Considering that there were more patients in the AC group with para-aortic MLNs, we conducted multivariate analysis and propensity score matching to verify the results. With multivariate analysis, AC was an independent prognostic factor of OS (*p* = 0.003) and DFS (*p* < 0.001). After propensity score matching at a ratio of 1:1, the 3-year OS rates were 86.4 and 75.4% (*p* = 0.017) in the SCC and AC groups, and the 3-year DFS rates were 82.5 and 57.3% (*p* = 0.001). Patients in the AC group had significantly worse survival.

A previous study suggested that the survival of patients with cervical AC/ASC was poor when treated with radiotherapy alone. With CCRT, the survival was similar between AC/ASC and SCC groups [7]. In our study, regardless of treatment with radiotherapy or CCRT, OS and DFS were significantly different between two groups. It should be noted that the HR values of DFS were 3.22 for patients receiving radiotherapy alone, and 1.97 for patients treated with CCRT. This may indicate that the survival difference between AC and SCC of the cervix became smaller with the introduction of concurrent chemotherapy.

The poor radio-sensitivity of cervical AC is one of the causes of worse survival of AC. It has been reported that patients with AC had poorer complete response (CR) and local control rates and required a longer time to achieve CR compared with patients with SCC after definitive radiotherapy or CCRT [15, 16, 18]. Similarly, patients with AC experienced more local failure in our study. We also found that patients with AC were more likely to have distant failure. This was significant in the comparison with log-rank method before (*p* = 0.011) and after matching (*p* = 0.009). In multivariate analysis, AC was an independent factor of distant control (*p* = 0.003). Even in patients with pelvic control, patients with AC still had poorer distant control (*p* = 0.027) and DFS (*p* = 0.009) than those with SCC. This indicated that poor radio-sensitivity was not the only the cause of worse survival of AC patients. Patients with AC also had a higher risk of distant recurrence after radiotherapy or CCRT.

Considering the poor survival of patients with AC of the cervix, we may need more effective protocols for these patients. One strategy is neoadjuvant or adjuvant chemotherapy. In a clinical trial from China, 880 patients with FIGO stage IIB-IVA cervical AC were randomised to receive either CCRT or CCRT with one cycle of neoadjuvant chemotherapy and two cycles of consolidation chemotherapy. The regimen for both neoadjuvant and consolidation chemotherapy were paclitaxel plus cisplatin. After a median follow-up period of 60 months, patients in the CCRT combined with neoadjuvant and adjuvant chemotherapy group experienced better DFS (*p* < 0.05), cumulative survival (< 0.05) and local control (*p* < 0.05). These results suggest that incorporating neoadjuvant and adjuvant chemotherapy to CCRT is a promising approach to improve the survival of patients with cervical AC [23]. Currently, cisplatin is the most favourable drug for concurrent chemotherapy. It is effective in the treatment of SCC of cervix. However, cisplatin may not be appropriate for patients with AC. Some studies have indicated that paclitaxel was an active agent for AC of the cervix [24, 25]. Huang et al. reported that, in cervical AC/ASC patients with advanced stage or MLNs, the 5-year relapse-free survival rates for patients treated with radiotherapy alone (45 patients), cisplatin-based CCRT (36 patients) and paclitaxel-based CCRT (13 patients) were 41.7, 41.7, and 53.8% (*p* = 0.611) [26]. In the present study, of the 60 AC patients treated with CCRT, 14 patients received paclitaxel/paclitaxel plus cisplatin. In subgroup analysis, the 3-year OS, DFS, pelvic control and distant control rates of AC patients treated cisplatin and paclitaxel-based regimens were 78.5 and 84.6% (*p* = 0.819), 54.0 and 85.1% (*p* = 0.163), 72.1 and 85.1% (*p* = 0.149) and 71.8 and 85.1% (*p* = 0.425), respectively. Paclitaxel trended toward improved survival, although the differences were not significant. These suggested the efficacy of paclitaxel in concurrent setting.

## Conclusion

The present study demonstrated that patients with AC of the cervix had poorer OS and DFS than patients with SCC, regardless of treatment with radiotherapy alone or CCRT. New treatment approaches should be considered for cervical AC.

## Abbreviations

AC: Adenocarcinoma
ASC: Adenosquamous carcinoma
CCRT: Concurrent chemoradiotherapy
CI: Confidence interval
CR: Complete response
CTV: Clinical target volume
DFS: Disease-free survival
FIGO: International Federation of Gynaecology and Obstetrics
GOG: Gynaecologic Oncology Group
GTV: Gross tumour volume
HR: Hazard ratio
ICBT: Intracavitary brachytherapy
IMRT: Intensity modulated radiation therapy
IRB: Institutional Review Board
MLNs: Metastatic lymph nodes
MVCT: Megavoltage computed tomography
OS: Overall survival
PCTV: Planning clinical target volume
PFS: Progression-free survival
PGTV: Planning gross tumour volume
SCC: Squamous cell carcinoma
